# Occurrence, identification, and antibiogram signatures of selected Enterobacteriaceae from Tsomo and Tyhume rivers in the Eastern Cape Province, Republic of South Africa

**DOI:** 10.1371/journal.pone.0238084

**Published:** 2020-12-07

**Authors:** Folake Temitope Fadare, Martins Ajibade Adefisoye, Anthony Ifeanyi Okoh

**Affiliations:** 1 SAMRC Microbial Water Quality Monitoring Centre, University of Fort Hare, Alice, South Africa; 2 Applied and Environmental Microbiology Research Group, Department of Biochemistry and Microbiology, University of Fort Hare, Alice, South Africa; Nitte University, INDIA

## Abstract

The increasing occurrence of multidrug-resistant Enterobacteriaceae in clinical and environmental settings has been seen globally as a complex public health challenge, mostly in the developing nations where they significantly impact freshwater used for a variety of domestic purposes and irrigation. This paper detail the occurrence and antibiogram signatures of the Enterobacteriaceae community in Tsomo and Tyhume rivers within the Eastern Cape Province, the Republic of South Africa, using standard methods. The average distribution of the presumptive Enterobacteriaceae in the rivers ranged from 1 × 10^2^ CFU/100ml to 1.95 × 10^4^ CFU/100ml. We confirmed 56 (70.8%) out of 79 presumptive Enterobacteriaceae isolated being species within the family Enterobacteriaceae through the Matrix-Assisted Laser Desorption Ionization Time of Flight technique. *Citrobacter*-, *Enterobacter*-, *Klebsiella* species, and *Escherichia coli* were selected (n = 40) due to their pathogenic potentials for antibiogram profiling. The results of the antibiotic susceptibility testing gave a revelation that all the isolates were phenotypically multidrug-resistant. The resistance against ampicillin (95%), tetracycline and doxycycline (88%), and trimethoprim-sulfamethoxazole (85%) antibiotics were most prevalent. The Multiple Antibiotic Resistance indices stretched from 0.22 to 0.94, with the highest index observed in a *C*. *freundii* isolate. Molecular characterisation using the PCR technique revealed the dominance of *bla*_TEM_ (30%; 12/40) among the eight groups of β-lactams resistance determinants assayed. The prevalence of others was *bla*_CTX-M_ genes including group 1, 2 and 9 (27.5%), *bla*_SHV_ (20%), *bla*_OXA-1-like_ (10%), *bla*_PER_ (2.5%), and *bla*_VEB_ (0%). The frequencies of the resistance determinants for the carbapenems were *bla*_KPC_ (17.6%), *bla*_GES_ (11.8%), *bla*_IMP_ (11.8%), *bla*_VIM_ (11.8%), and *bla*_OXA-48-like_ (5.9%). Out of the six plasmid-mediated AmpC (pAmpC) genes investigated *bla*_ACC_, *bla*_EBC_, *bla*_FOX_, *bla*_CIT_, *bla*_DHA_, and *bla*_MOX_, only the first four were detected. In this category, the most dominant was *bla*_EBC_, with 18.4% (7/38). The prevalence of the non-β-lactamases include *tetA* (33.3%), *tetB* (30.5%), *tetC* (2.8%), *tetD* (11.1%), *tetK* (0%), *tetM* (13.9%), *catI* (12%), *catII* (68%), *sulI* (14.3%), *sulII* (22.9%) and *aadA* (8.3%). Notably, a *C*. *koseri* harboured 42.8% (12/28) of the genes assayed for which includes five of the ESBL genes (including the only *bla*_PER_ detected in this study), two of the pAmpC resistance genes (*bla*_ACC_ and *bla*_CIT_), and five of the non-β-lactamase genes. This study gives the first report on *C*. *koseri* exhibiting the co-occurrence of ESBL/AmpC β-lactamase genes from the environment to the best of our knowledge. The detection of a *bla*_PER_ producing *Citrobacter* spp. in this study is remarkable. These findings provide evidence that freshwater serves as reservoirs of antimicrobial resistance determinants, which can then be easily transferred to human beings via the food chain and water.

## Introduction

Globally, the appearance, widespread, and distribution of antimicrobial resistance in bacteria have been described as a complex public health challenge [1–3]. Initially, it was perceived as a problem restricted to clinical settings [4]. However, findings have shown that genes encoding various antibiotic resistance within the environment predate the discovery of antibiotics used in clinical settings. This discovery implicates the environment as the origin of antimicrobial resistance evolution [5–8]. In different regions of the world, various studies have investigated the occurrence of antibiotic-resistant bacteria (ARB) in a diverse aquatic milieu [5, 9–12]. Since the first discovery of antibiotics over eight decades ago, they have saved millions of lives in treating bacterial infections and diseases. However, this feat was short-lived as the numbers of organisms becoming resistant to antibiotics have grown at alarming rates than the anticipated normal evolutionary process for microorganisms [2, 4, 13]. Under certain circumstances, the ARB could be selected, such as when soil organisms that naturally can produce antibiotics are deposited to freshwater sources via runoffs. These natural stockpiles of ARB alongside the antibiotic-resistant genes (ARGs) conferring resistance on them could then be a wellspring of transferable traits for emerging pathogens.

Once individual organisms in a particular niche have developed or acquired resistance, they quickly transfer this to other organisms in widely varied niches. Various horizontal gene transfer mechanisms often facilitate this widespread, leading to the spread of resistant organisms [13–15]. Selective pressure favours the evolution of resistance occurs whenever antimicrobials are utilised in animal husbandry and aquaculture, as therapeutics, prophylaxis, metaphylaxis, and growth promoters [16–20]. This is primarily because, by nature, antibiotics act on the principle of selective toxicity wherein they kill or inhibit microbial pathogens while little or no damage is done to the host [2, 5, 16].

The gastrointestinal tract of animals and humans may serve as an alternate host or passive carrier of ARB, and these can cause different diseases via diverse mechanisms. When antibiotics are applied, ARB survives and multiply rapidly, reaching high densities in the intestinal lumen. They are then excreted into the environment, making the possibility of containing their spread far-fetched [21]. The ARB may initially be commensals living in the gastrointestinal tract, but after a while, may acquire genetic materials such as plasmids, integrons, or transposons carrying various resistance genes and virulence factors. The acquisition of these genetic materials can then transform these initially harmless bacteria into more virulent and resistant organisms [4, 16]. Also, many antibiotics used are not broken down into inactive constituents, thereby retaining their properties even after discharge from the body into the environment [4, 18, 22, 23]. These sometimes travel to wastewater treatment plants (WWTPs) via domestic sewer lines or are excreted directly into the soil or surface waters, thereby further adding to the antibiotic resistance pressure impacted on the environment [24, 25]. Surface waters have been reported to be ‘hotspots’ of antimicrobial resistance contamination due to being a recipient of discharges from diverse sources, including contaminants from industrial, agricultural, and domestic settings of varying chemical and microbial concerns [5, 18, 26, 27]. Furthermore, the surface water being a reservoir of ARB and ARGs could serve as a dissemination port for the proliferation of new resistant strains, which can be enhanced by acquiring bacteriophages or integrons through horizontal gene transfer mechanisms [28, 29].

One of the most prestigious groups of microorganisms that have been popularly implicated in the spread of ARGs in the environment is the Enterobacteriaceae group. Members of this family are among the natural microflora in the gastrointestinal tracts of warmblooded animals, including humans, and commonly found in diverse environmental sources such as water, plants, and soil [16]. Clinically significant genera of this family that commonly cause infections are *Citrobacter*-, *Enterobacter*-, *Escherichia coli*, *Klebsiella*-, *Morganella*-, *Plesiomonas*-, *Proteus*-, *Providencia*-, *Salmonella*-, *Serratia*-, *Shigella*- and *Yersinia* species [16, 30, 31]. They have been commonly implicated in various infections including pneumonia, enteritis, diarrhoea, septicaemia, wound and infections involving the central nervous system [16].

Various ARGs against various classes of antibiotics such as aminoglycosides, tetracyclines, sulphonamides, trimethoprim, β-lactams, and carbapenems have been discovered in multiple aquatic environments [7, 9, 10, 12]. The principal mechanism of antibiotic resistance in the family Enterobacteriaceae is the production of enzymes called β-lactamases. These enzymes include the Extended-Spectrum β-lactamases (ESBLs) and the plasmid-mediated AmpC-producing (pAmpC) [32]. The β-lactams have a particular chemical structure called the β-lactam ring and are the largest and most generally used antibiotics class. These are mostly semi-synthetic compounds, which emanate from bacteria and fungi within the environment [33, 34]. They function by preventing the transpeptidation of the peptidoglycan cell wall of the invading bacteria, which is necessary to complete the cell wall [33–35]. Resistance against the β-lactams is principally by the structural modification of the penicillin-binding proteins, thereby resulting in the decreased attraction of the drugs or, worse still, the bacteria produce enzymes, which break the β-lactam ring, thereby rendering the drugs ineffective. Other resistance mechanisms include decreased permeability or active transportation of the antibiotics out of the bacterial cell using efflux pumps [35].

The ability of Enterobacteriaceae species to produce enzymes ESBL and pAmpC is a severe emerging problem and a source of concern for environmental safety [1, 36–38]. These enzymes endow bacteria with various resistances against most β-lactam antibiotics, including penicillins, carbapenems, and cephalosporins [16, 34, 39]. The ability to produce the ESBL/pAmpC enzymes that cleave the β-lactam ring, thereby conferring resistance on the organisms, makes it one of the most potent ways of fostering resistance in the Enterobacteriaceae family [38]. This scenario thereby undermines current antibiotics’ effectiveness and, unfortunately, further hampers new drugs’ development. Initially, carbapenems were prescribed as a last resort for the therapy of gram-negative severe nosocomial infections caused by ESBLs and pAmpC producing Enterobacteriaceae. Unfortunately, due to this class of antibiotics’ widespread use, various carbapenem-resistant Enterobacteriaceae has been reported [40, 41]. The ARB can be readily spread directly to humans when contaminated water is consumed or indirectly via the consumption of foods irrigated with water from these contaminated freshwater sources. This further enables the spread and persistence of ARB and ARGs within our environment and the general populace [42, 43]. Therefore, studies must be carried out to monitor the prevalence of ARB and ARGs in freshwater sources. Here, we describe the Enterobacteriaceae community’s occurrence and antibiogram signatures in Tsomo and Tyhume rivers in ECP, RSA.

## Materials and methods

### Study sites

Two freshwater resources sampled in this study are the Tyhume River and Tsomo River. The Tyhume River is found in Amathole District Municipality (ADM), while the Tsomo River is located in Chris Hani District Municipality (CHDM), both in the ECP. In this province, ADM is located in the central part, while CHDM is situated in the northeastern region. They are both mainly involved in agricultural production and serve as a hub of various agricultural processes, which may be due to their nearness to East London and Port Elizabeth seaports.

The Tyhume River is situated in the Raymond Mhlaba local municipality within ADM. The river originates from the mountains in Hogsback and passes through several rural settlements along its path to Alice, and finally empties to the Kieskamma River at Manqulweni community. This river’s different host communities are using it for diverse domestic activities such as washing, drinking, cooking, recreational activities, and religious activities, including baptism, especially in locations where potable water is not within reach. It is also channeled into the Binfield Park Dam, a hub for supplying raw water to the different water treatment plants in that vicinity. The treated water is then subsequently redistributed to Alice town and the communities in that area as a source of potable water. The four sampling sites along the Tyhume River include Hala, Khayalethu, Sinakanaka, and Alice.

**Fig 1:**
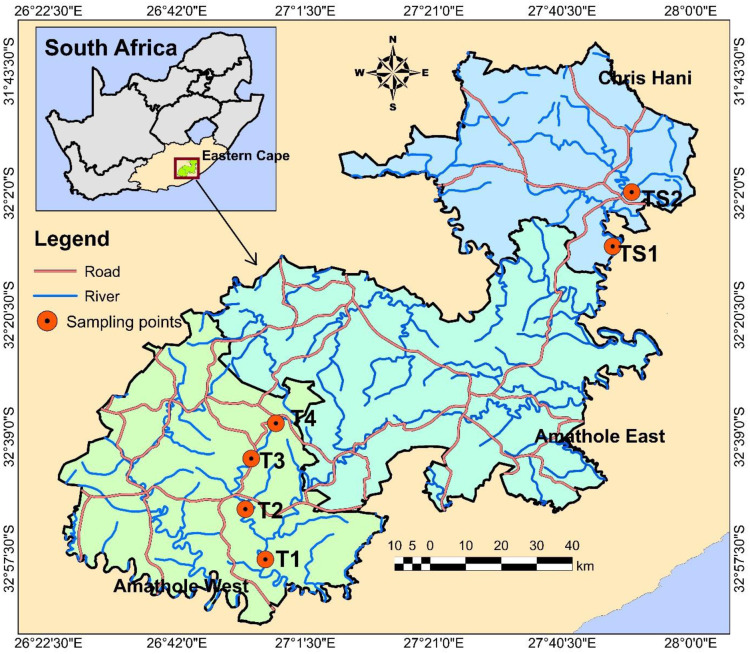
Location of sampling sites in Tyhume and Tsomo River. Site codes TS1 represents Bongweni, and TS2 represents Tsomo, which are sampling points along the Tsomo river in Chris Hani District Municipality. Site codes T1 –T4 represents Hala, Khayalethu, Sinakanaka, and Alice, respectively, which are sampling points along the Tyhume river in Amathole District Municipality.

The Tsomo River is found in the Intsika Yethu local municipality within CHDM. This river originates about 10 km to the northwest of the town Elliot and flows through several rural settlements, including Tsomo, Cala, and Ncora. It flows southwards and finally empties into the Great Kei River. The different host communities also use this river it passes through for various domestic purposes. The river is channeled into the Ncora Dam, which is used solely for irrigation purposes. The details of these sample collection points and their geographic coordinates are described in the S1 Table in S1 File. The map showing the sample collection sites is shown in Fig 1.

### Sample collection and processing

In October and November 2017, water samples were collected from four and two points on the Tyhume and Tsomo River, respectively, in triplicates on a once-off basis. With the aid of sterile 1 L polypropylene bottles, samples were retrieved from each site in triplicates. These were maintained at 4°C and immediately transported to the laboratory and analysed within 6 hours. Ten-fold serial dilution (10^−1^, 10^−2^, and 10^−3^) was carried out, then 100 mL of the appropriately diluted sample was filtered through a cellulose nitrate membrane filter with a pore size of 0.45 μm (Sartorius, Goettingen, Germany) using a vacuum pump. All samples were processed in triplicates. After each filtration, the membrane filter was aseptically placed onto Petri-dishes containing sterile Violet Red Bile Glucose (VRBG) agar (Conda—pronadisa, USA) plates incubated at 37°C for 24 hours for the enumeration and isolation of presumptive Enterobacteriaceae. After incubation, purple-red colonies with a diameter of at least 0.5 mm were counted as characteristic presumptive Enterobacteriaceae. The results obtained were recorded as colony-forming units per 100 ml (CFU /100ml) [44].

### Isolation and MALDI-TOF identification of presumptive targeted Enterobacteriaceae

The isolation was executed following standard procedures utilising general and selective media. Morphologically different colonies were sub-cultured on sterile Eosin Methylene Blue differential agar (EMB) (Merck, Darmstadt, Germany) and incubated at 37°C for 24 hours. Green metallic sheen colonies and pink to dark purple colonies were selected as lactose fermenters (presumptive *E*. *coli*, *Citrobacter-*, *Enterobacter-* and *Klebsiella* species) purified using nutrient agar. Pure presumptive isolates were preserved in 20% glycerol stock solutions at -80°C for future assays. The confirmation of isolates’ identification was obtained by using matrix-assisted laser desorption and ionisation time-of-flight coupled with mass spectrometry (MALDI-TOF MS). The MALDI Biotyper 3.0 software (Bruker Daltonics, Bremen, Germany) was used to confirm the identities of the presumptive organisms to species level. The instrument was calibrated with bacterial test standard (BTS), and all analysis was carried out in duplicates. All results were interpreted following the manufacturer’s instructions, and results with values less than 1.700 were excluded as their identification was not reliable as described by [45].

### Antibiotic susceptibility testing

The antimicrobial susceptibility patterns of all targeted identified Enterobacteriaceae isolates totaling 40 recovered from the rivers were analysed using the Kirby-Bauer disk diffusion technique following the Clinical and Laboratory Standards Institute guidelines [46]. The isolates were subjected to 18 antibiotic discs that belong to 11 different classes of antibiotics which are usually used to treat infections triggered by Enterobacteriaceae, as suggested by the Center for Disease Control and Prevention (CDC). The antibiotic classes, the specific antibiotics used along with their concentrations are as follows; Aminoglycosides: gentamicin (GM; 10 μg) and amikacin (AK; 30 μg); β-lactams: amoxicillin/clavulanic acid (AUG; 30 μg) and ampicillin (AP; 10 μg); Carbapenems: imipenem (IMI; 10 μg) and meropenem (MEM; 10 μg); Cephems: cefotaxime (CTX; 30 μg) and cefuroxime (CXM; 30 μg); Fluoroquinolones: ciprofloxacin (CIP; 5 μg) and norfloxacin (NOR: 30 μg); Nitrofurans: nitrofurantoin (NI; 300 μg); Phenicols: chloramphenicol (C; 30 μg); Polymyxins: polymyxin B (PB; 300 units) and colistin sulphate (CO; 25 μg); Quinolones: nalidixic acid (NA; 30 μg); Sulfonamides: trimethoprim-sulfamethoxazole (TS; 25 μg); Tetracyclines: tetracycline (T; 30 μg) and doxycycline (DXT; 30 μg). In brief, the inoculum was prepared in normal saline solution by dispersing a single colony picked with a sterile cotton swab. The resulting solution’s turbidity was compared to 0.5 McFarland turbidity standards (equivalent to 1.5 × 10^8^) and, where necessary, adjusted using sterile normal saline. Then, 100 μl of each standardised bacterial test suspension was spread on the Mueller-Hinton agar (Merck, Johannesburg) plates using sterile cotton swabs. Using a disc dispensing apparatus (Mast Diagnostics, U.K), the relevant antibiotic disks (Mast Diagnostics, U.K) were placed at an equidistance of 30 mm on the inoculated plates. The plates were then inverted fifteen minutes after the discs were applied and incubated at 37°C for 18–24 hours. The inhibition zones were determined by measuring the clear zones’ diameter around the antibiotic disks to the nearest millimeters. The results for the isolates were interpreted as "Resistant (R), Intermediate (I), or Susceptible (S)" following the [46] breakpoints. In contrast, the class Polymyxins were interpreted using the zone diameter of *E*. *coli* ATCC 25922.

### Analysis of Multiple Antibiotic Resistance Phenotype (MARP) and Multiple Antibiotic Resistance Index (MARI) of bacterial isolates

The MARP patterns for isolates that displayed resistance against more than three antibiotics out of the tested eighteen were assessed [47]. Isolates that were resistant against at least three antimicrobial classes were classified as multidrug-resistant (MDR). The multidrug resistance pattern, the number of antibiotics the isolates displayed resistance against, and the number of observed phenotypic patterns were analysed. The individual multidrug isolate’s MARI was derived using a Mathematical equation described by Krumperman [48]. The mathematical expression is given as:

MARindex=x/y

Where “x” is the number of antibiotics against which a bacterial species exhibited resistance, ''y'' is the total number of antibiotics that bacterial species were exposed to. A MAR index value of more than 0.2 is an indicator of intensive usage of antibiotics in that area and predict high-risk vicinity for the possible promotion of antibiotic resistance [48, 49].

### Antibiotic resistance genes detection

Purified bacteria isolates were revived by inoculating into a nutrient broth and then incubated overnight at 37°C. The cultures were then transferred unto nutrient agar (N.A.) and incubated overnight at 37°C. The total genomic DNA extraction was done through the boiling method, as reported by [50] with slight modifications. Briefly, with the aid of a sterilised inoculating loop, single colonies from N.A. were suspended into 100 μl of sterilised nuclease-free water (Thermo Scientific, USA) in sterile Eppendorf tubes (Biologix, USA). The resulting suspension was swirled with a vortex mixer (Digisystem Laboratory, Taiwan). Boiling at 100°C for 10 minutes with an AccuBlock (Digital dry bath, Labnet) lysed the cells. These were incubated immediately on ice for 5 mins; after that, the cell debris was extracted by centrifuging at 13,500 rpm for 10 minutes using a Mini-spin micro-centrifuge (LASEC, RSA). Cell lysate supernatant was removed into sterile Eppendorf tubes, which were utilised as template DNA in PCR assays straight away or stowed at -20°C for future assays. Different resistance determinants were assayed for in the targeted bacterial isolates showing full or intermediate resistance. Nineteen resistance genes that encode β-lactamases, various variants of ESBL, and pAmpC resistance gene determinants were assayed for in duplex and multiplex PCR protocols, as reported by [51]. Each gene with the primer set used, and their expected molecular sizes are described in S2 Table in S1 File. The thermal cycling conditions for the PCR assays were as follows: initial denaturation at 94°C for 10 mins; followed by 30 cycles of 94°C for 40s, 60°C for 40s, and 72°C for 1 min; and then a final elongation step at 72°C for 7 mins. For the carbapenemase genes (*bla*_VIM_, *bla*_IMP_, and *bla*_KPC_) and (*bla*_GES_ and *bla*_oxa-48-like_) multiplex PCR assays, the annealing temperature was optimal at 55°C and 57°C, respectively. Singleplex, duplex, and multiplex PCR protocols, as reported by [9], were employed to detect 12 resistance genes that code for resistance against non-β-lactams, including the sulphonamides, tetracyclines, phenicols, and aminoglycosides. S3 Table in S1 File shows the list of the genes assayed with their expected molecular sizes.

For the PCR assays, a Mycycler^™^ Thermal Cycler PCR system (BioRad, USA) was used. Each of the reaction composed of 12.5 μl double strength master mix (Thermo Scientific, USA), 1 μl each of the primers synthesised by Inqaba Biotech (Pretoria, RSA), 5.5 μl of nuclease-free water (Thermo Scientific, USA), and DNA template in a total reaction volume of 25 μl. Negative controls were used in all reactions, which consisted of all the reaction mixture above except that the DNA template was replaced with PCR buffer and nuclease-free water. Each amplified DNA (5 μl) was loaded in a 1.5% (w/v) horizontal agarose gel (Separations, South Africa) containing Ethidium Bromide (0.001 μg/ml). A DNA ladder of either 50 or 100-bp (Thermo Scientific, USA) was also added to each gel during electrophoresis to estimate the appropriate expected band size for each of the genes assayed for depending on availability. The gels ran using 0.5X TBE buffer for 45 mins at 100 volts and visualised by a U.V. transillumination (ALLIANCE 4.7).

### Data analysis

One-way analysis of variance (ANOVA) with a 95% confidence interval was carried out to test the Enterobacteriaceae counts’ significant difference across the different sampling sites. The antibiotic susceptibility test results were analysed using Microsoft Excel 2010.

## Results

### The distribution of Enterobacteriaceae in river water

**Fig 2:**
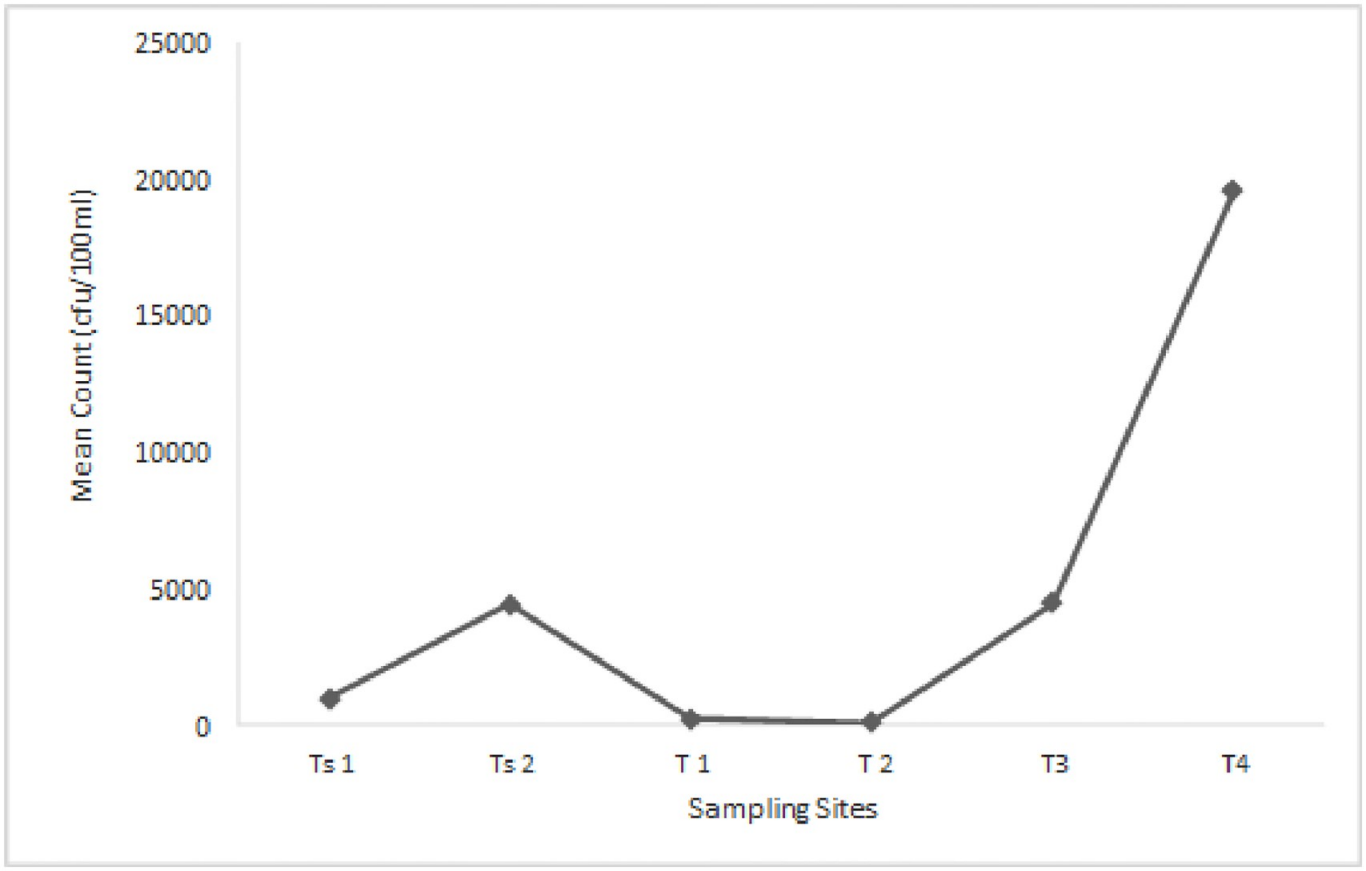
The mean of Enterobacteriaceae cell counts from Tsomo and Tyhume rivers. Ts1 and Ts2 are sampling points along the Tsomo river, while T1 –T4 are sampling points along the Tyhume river. Using the Welch and Brown-Forsythe robust tests of equality of means, the mean counts were statistically significant at *P* ≤0.05 and F = 256.59. The statistics were asymptotically F distributed.

The distribution of presumptive Enterobacteriaceae in the rivers ranged from 1 × 10^2^ cfu/100ml in T2 to 1.95 × 10^4^ cfu/100ml in T4, as shown in Fig 2. The distributions of Enterobacteriaceae in rivers collected from both District Municipalities were statistically significant at *P≤* 0.05.

### MALDI-TOF identification of presumptive Enterobacteriaceae

**Fig 3:**
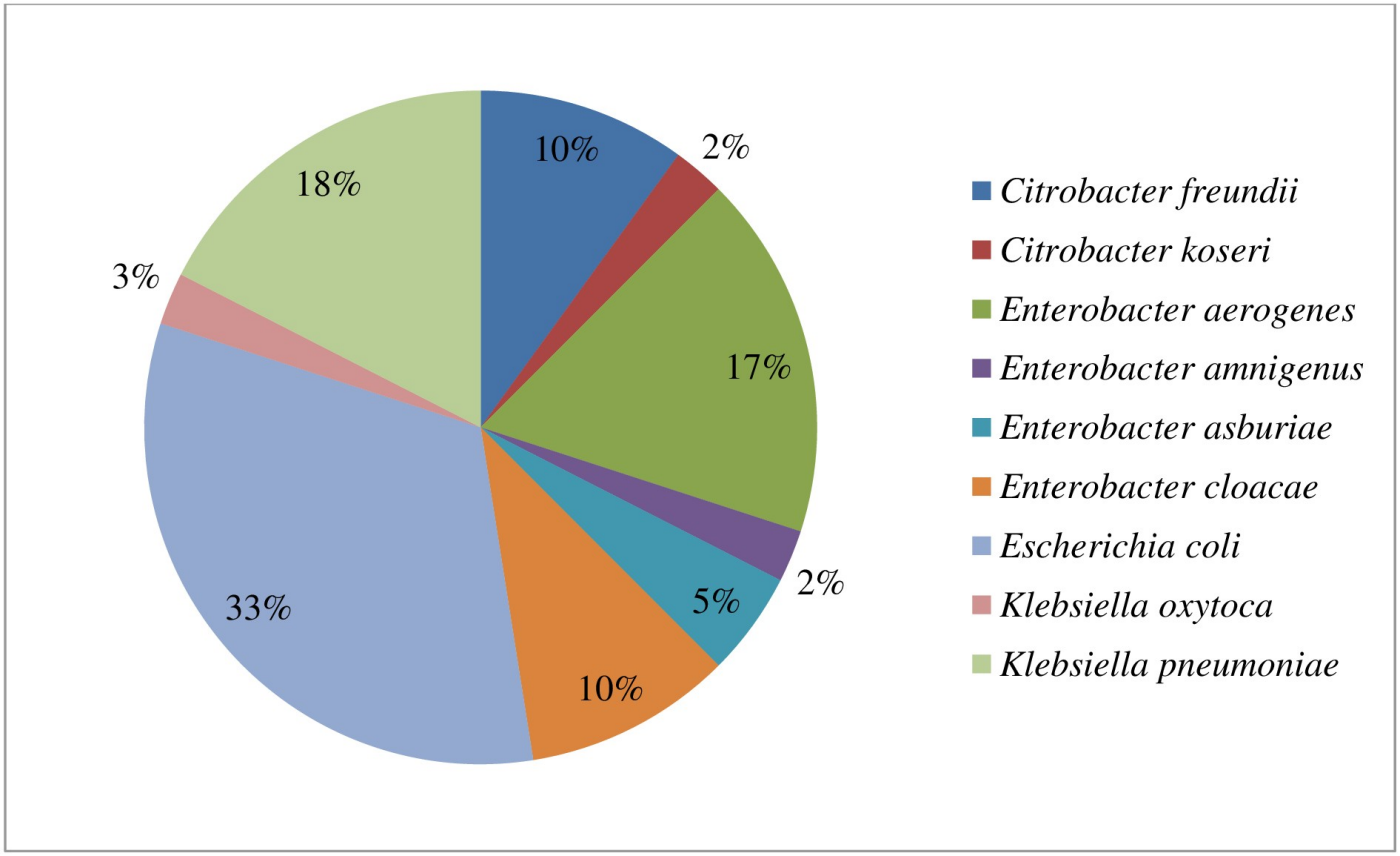
The distribution of the confirmed members of the targeted Enterobacteriaceae (n = 40) recovered from the rivers.

Out of the 79 presumptive Enterobacteriaceae isolates recovered from the rivers, 56 were confirmed as Enterobacteriaceae members. They belonged to seven genera with *Citrobacter-*, *Enterobacter-*, *Escherichia-*, *Klebsiella-*, *Plesiomonas-*, *Proteus-* and *Serratia*. Other bacteria families identified include Bacillaceae (2), Morganellaceae (1), Pseudomonadaceae (5), and Staphylococcaceae (2). Thirteen of the isolates had a MALDI-TOF score value less than 1.7, and as such, their identities were not reliable and consequently excluded from this study. The isolates’ full identification showing the various genus and species identified, their numbers observed, and the families they belong to are presented in S4 Table in S1 File. The total numbers of targeted members of the Enterobacteriaceae family (*Citrobacter* species, *Enterobacter* species, *Klebsiella* species, and *Escherichia coli*) considered in this study were 40. Among this bacterial species, *E*. *coli* was dominantly isolated, comprising 33%, followed by *K*. *pneumoniae* and *E*. *aerogenes*, 18% and 17%, respectively, of the total isolates. The distribution of the bacteria isolates is shown in Fig 3.

### Evaluation of antibiotic susceptibility profile of targeted members of Enterobacteriaceae to a panel of test antibiotics

**Fig 4:**
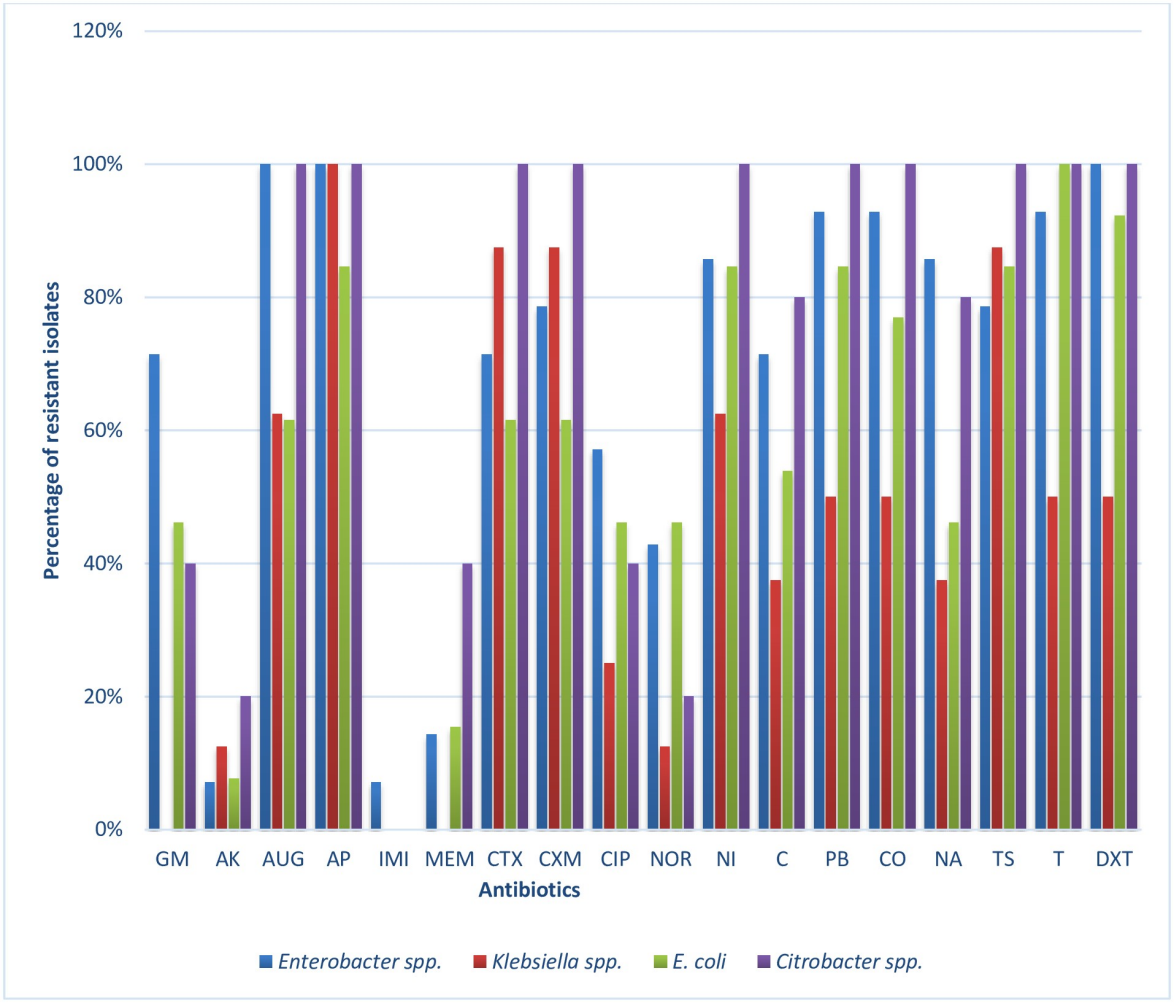
The antibiotic resistance frequencies of targeted Enterobacteriaceae recovered from Tyhume and Tsomo Rivers. *Enterobacter* spp. (n = 14), *Klebsiella* spp. (n = 8), *Citrobacter* spp. (n = 5) and *E*. *coli* (n = 13). Antibiotics code: GM-Gentamicin, AK-Amikacin, AUG—Amoxicillin/Clavulanic acid, AM- Ampicillin, IMI- Imipenem, MEM- Meropenem, CTX-Cefotaxime, CXM-Cefuroxime CIP-Ciprofloxacin, NOR-Norfloxacin, NI-Nitrofurantoin, C-Chloramphenicol, PB- Polymyxin B, CO-Colistin sulphate, TS-Trimethoprim/Sulfamethoxazole, T-Tetracycline, and DXT-Doxycycline.

The antibiotic resistance profiles of the confirmed targeted members of Enterobacteriaceae (n = 40) recovered from the rivers are shown in Fig 4. All the 14 *Enterobacter* spp. isolated exhibited resistance against the two selected antibiotics in the β-lactams (ampicillin and amoxicillin/clavulanic acid) as well as doxycycline. This was closely followed by resistance against the two polymyxins (polymyxin B and colistin sulphate) and tetracycline, where 93% of the isolates exhibited phenotypic resistance against these three antibiotics. The antibiotics to which the least resistance was observed were amikacin and imipenem with 7%.

For *Klebsiella* species, all the eight isolates exhibited resistance against ampicillin, followed by the cephems class (cefotaxime and cefuroxime) and trimethoprim-sulfamethoxazole with a resistance frequency of 88%. No resistance was observed against gentamicin, imipenem, and meropenem amongst all the *Klebsiella* spp. from the freshwater sources. The two highest frequency of resistance observed in *E*. *coli* isolates to antibiotics were recorded for the class tetracyclines wherein they were all resistant against tetracycline. At the same time, 92% exhibited resistance against doxycycline. This was followed by the resistance of 85% displayed across four different classes of antibiotics, including antibiotics ampicillin, nitrofurantoin, polymyxin B, and trimethoprim-sulfamethoxazole. It is interesting to note that all five *Citrobacter* spp. which were isolated, displayed resistance against at least 10 of the 18 tested antibiotics. These resistances were displayed across six different classes of antibiotics. However, no resistance was observed to imipenem as all the isolates were susceptible to this carbapenem.

### Phenotypic antibiotic resistance pattern of targeted members of Enterobacteriaceae from rivers

**Fig 5:**
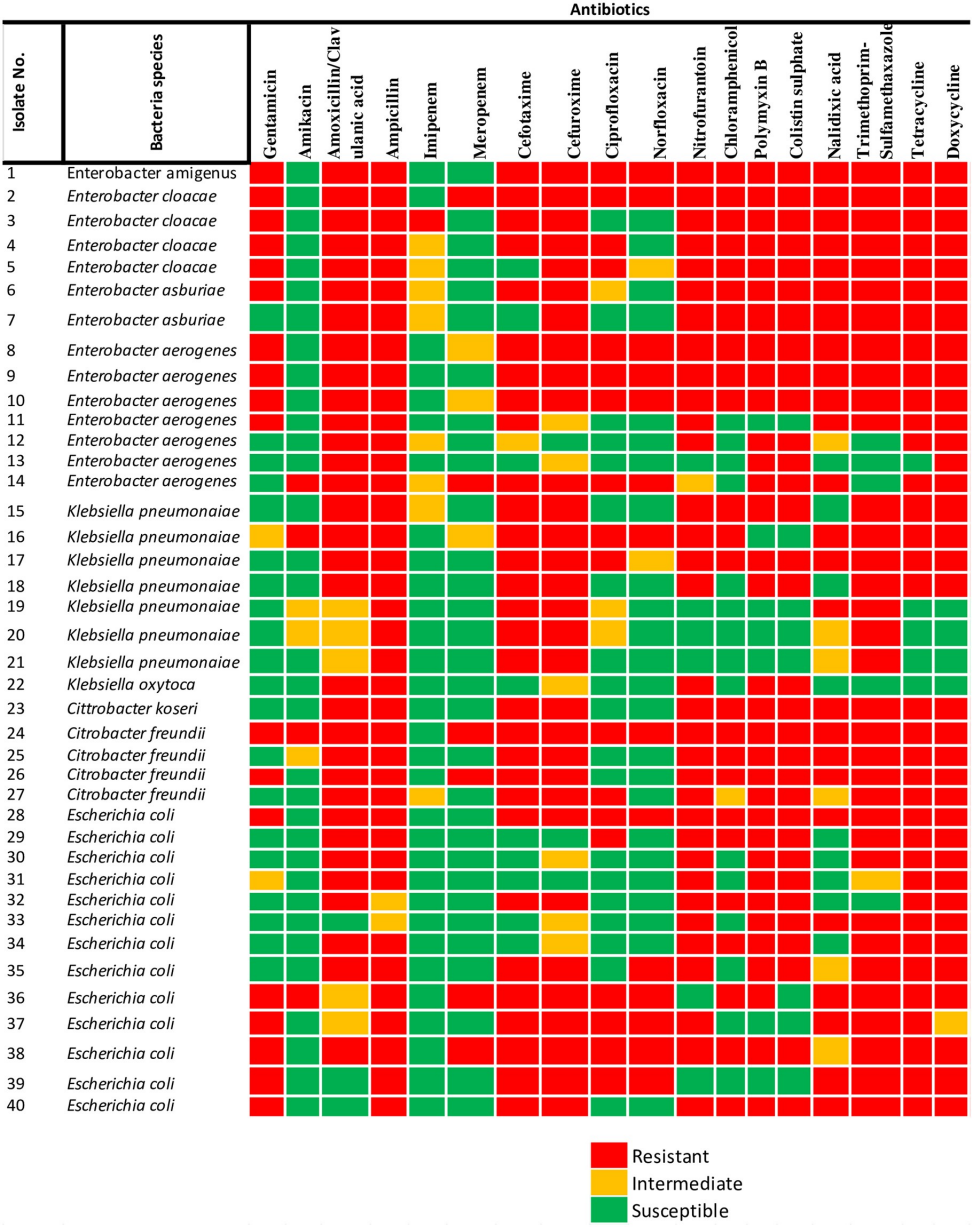
Members of Enterobacteriaceae isolated from rivers, indicating the phenotypic antibiotic resistance profiles. The colour codes indicate resistant, intermediate, and susceptible phenotypes to specific eighteen antibiotics belonging to eleven classes.

Generally, varying phenotypic resistance pattern was recorded for most of the targeted members of Enterobacteriaceae recovered. The antibiotic susceptibility pattern for each bacteria isolates to each tested antibiotics is shown in Fig 5. The result observed indicates the antibiotics’ effectiveness towards each isolate, which indicates the probable genetic characteristics possessed by each bacteria species. From this result, all the isolates showed full resistance against ampicillin except for isolates 32 and 33, which showed intermediate resistance. One *C*. *freundii* (isolate No. 24) displayed resistance phenotypically against all the test antibiotics except for imipenem.

### Assessment of MARP and MARI of targeted members of the Enterobacteriaceae group

The MARP patterns with the MARI exhibited by *Enterobacter* spp. and *Klebsiella* spp. are shown in Table 1, while those of *Citrobacter* spp. and *E*. *coli* are shown in Table 2. All the targeted bacteria recovered displayed resistance against at least four of the test antibiotics. In contrast, the highest resistance pattern was observed in a *C*. *freundii*, which was resistant against 17 out of the 18 tested antibiotics. The MARI generally ranged from 0.22 to 0.94 amongst all the isolates, higher than the permissible MARI value of 0.2. The majority of the phenotypes were observed uniquely.

**Table 1:** MAR phenotypes and MAR indices patterns in *Enterobacter* spp. and *Klebsiella* spp. isolated from the rivers.

*Enterobacter* species (n = 14)			
MAR phenotypes^a^	No of antibiotics showing resistance	No of phenotype observed	MAR index
GM-AUG-AP-MEM-CTX-CXM-CIP-NOR-NI-C-PB-CO-NA-TS-T-DXT	16	1	0.89
GM-AUG-AP-CTX-CXM-CIP-NOR-NI-C-PB-CO-NA-TS-T-DXT	15	4	0.83
GM-AUG-AP-IMI-CTX-CXM-NI-C-PB-CO-NA-TS-T-DXT	14	1	0.78
GM-AUG-AP-CTX-CXM-CIP-NI-C-PB-CO-NA-TS-T-DXT	14	1	0.78
GM-AUG-AP-CTX-CXM-NI-C-PB-CO-NA-TS-T-DXT	13	1	0.72
AK-AUG-AP-MEM-CTX-CXM-CIP-NOR-PB-CO-NA-T-DXT	13	1	0.72
AUG-AP-CXM-CIP-NI-C-PB-CO-NA-TS-T-DXT	12	1	0.67
AUG-AP-CXM-NOR-NI-C-PB-CO-NA-TS-T-DXT	12	1	0.67
GM-AUG-AP-CTX-NI-NA-TS-T-DXT	9	1	0.50
AUG-AP-NI-PB-CO-T-DXT	7	1	0.39
AUG-AP-PB-CO-DXT	5	1	0.28
***Klebsiella* species (n = 8)**			
AK-AUG-AP-CTX-CXM-CIP-NOR-NI-C-NA-TS-T-DXT	13	1	0.72
AUG-AP-CTX-CXM-CIP-NI-C-PB-CO-NA-TS-T-DXT	13	1	0.72
AUG-AP-CTX-CXM-NI-C-PB-CO-TS-T-DXT	11	1	0.61
AUG-AP-CTX-CXM-NI-PB-CO-TS-T-DXT	10	1	0.56
AUG-AP-NI-PB-CO	5	1	0.28
AP-CTX-CXM-NA-TS	5	1	0.28
AP-CTX-CXM-TS	4	2	0.22

^**a**^ represents antibiotics code which are explained in Fig 4 footnotes.

**Table 2:** MAR phenotypes and MAR indices patterns in *Citrobacter* spp. and *E*. *coli* isolated from the rivers.

*Citrobacter* species (n = 5)			
MAR phenotypes^a^	No of antibiotics showing resistance	No of phenotype observed	MAR index
GM-AK-AUG-AP-MEM-CTX-CXM-CIP-NOR-NI-C-PB-CO-NA-TS-T-DXT	17	1	0.94
GM-AUG-AP-MEM-CTX-CXM-NI-C-PB-CO-NA-TS-T-DXT	14	1	0.78
AUG-AP-CTX-CXM-NI-C-PB-CO-NA-TS-T-DXT	12	2	0.67
AUG-AP-CTX-CXM-CIP-NI-PB-CO-TS-T-DXT	11	1	0.61
***E*. *coli* (n = 13)**			
GM-AUG-AP-MEM-CTX-CXM-CIP-NOR-NI-C-PB-CO-TS-T-DXT	15	1	0.83
GM-AUG-AP-CTX-CXM-CIP-NOR-NI-C-PB-CO-NA-TS-T-DXT	15	1	0.83
GM-AK-AP-MEM-CTX-CXM-CIP-NOR-C-PB-NA-TS-T-DXT	14	1	0.78
GM-AP-CTX-CXM-NI-C-PB-CO-NA-TS-T-DXT	12	1	0.67
AUG-AP-CTX-CXM-NOR-NI-PB-CO-TS-T-DXT	11	1	0.61
GM-AP-CTX-CXM-CIP-NOR-NI-NA-TS-T	10	1	0.56
GM-AP-CTX-CXM-CIP-NOR-NA-TS-T-DXT	10	1	0.56
AUG-AP-CIP-NI-C-PB-CO-TS-T-DXT	10	1	0.56
AUG-AP-NI-C-PB-CO-TS-T-DXT	9	1	0.50
AUG-CTX-CXM-NI-C-PB-CO-T-DXT	9	1	0.50
AUG-AP-NI-PB-CO-TS-T-DXT	8	1	0.44
AUG-AP-NI-PB-CO-T-DXT	7	1	0.39
NI-PB-CO-NA-TS-T-DXT	7	1	0.39

^**a**^ represents antibiotics code which are explained in Fig 4 footnotes.

### The assortment of antibiotic resistance genes (ARGs) in the Enterobacteriaceae species

**Fig 6:**
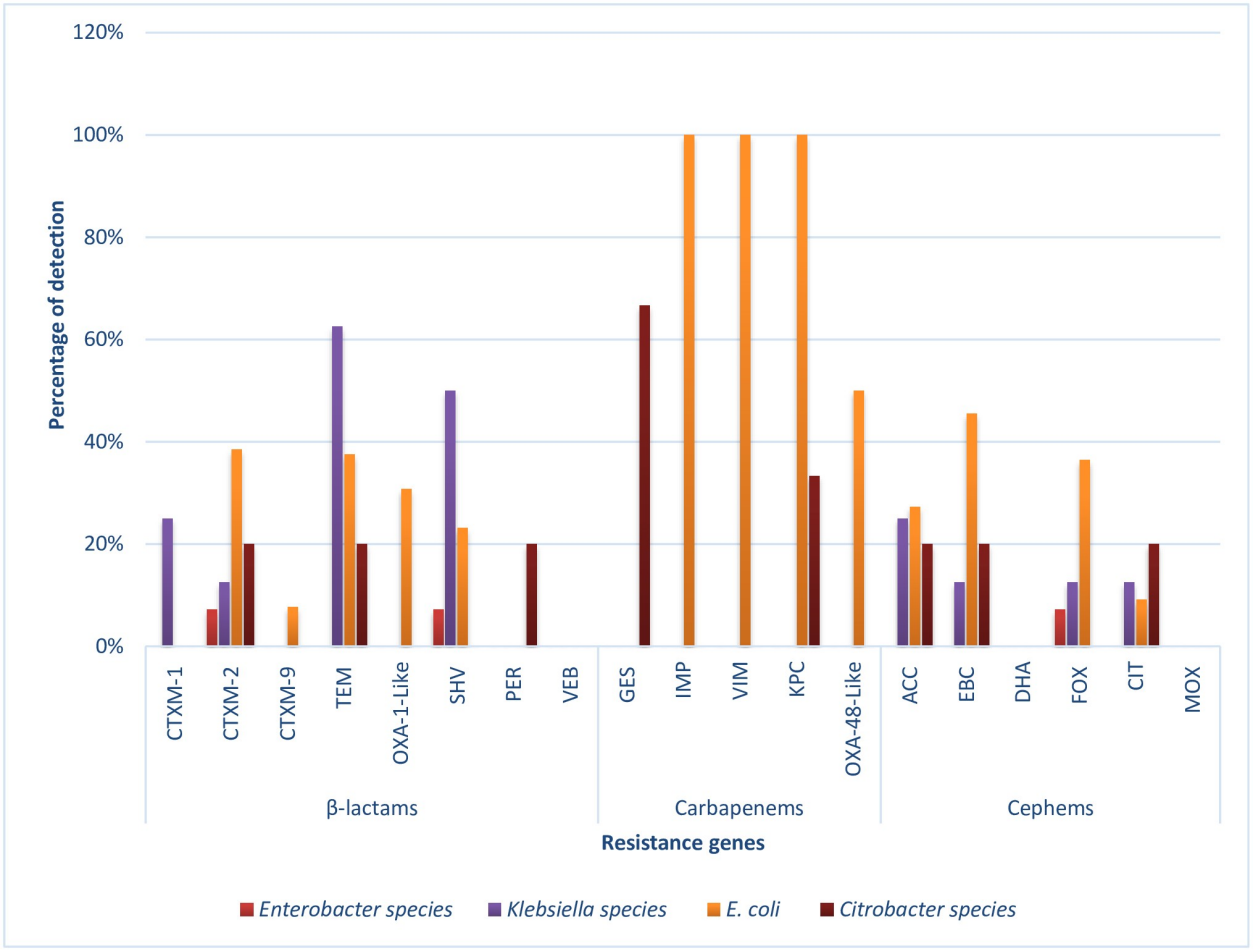
The prevalence of key β-lactamases genes detected across the bacterial isolates. These include the pAmpC genes coding for resistance against the cephems class of antibiotics and other ESBL resistance encoding genes. For β-lactams: *Enterobacter* spp. (n = 14), *Klebsiella* spp. (n = 8), *E*. *coli* (n = 13) and *Citrobacter* spp (n = 5); Carbapenems: *Enterobacter* spp. (n = 10), *Klebsiella* spp. (n = 2), *E*. *coli* (n = 2) and *Citrobacter* spp. (n = 3); Cephems: *Enterobacter* spp. (n = 14), *Klebsiella* spp. (n = 8), *E*. *coli* (n = 11) and *Citrobacter* spp. (n = 5), where n is the number of phenotypic resistant isolates screened for the representative antibiotic resistance genes.

**Fig 7:**
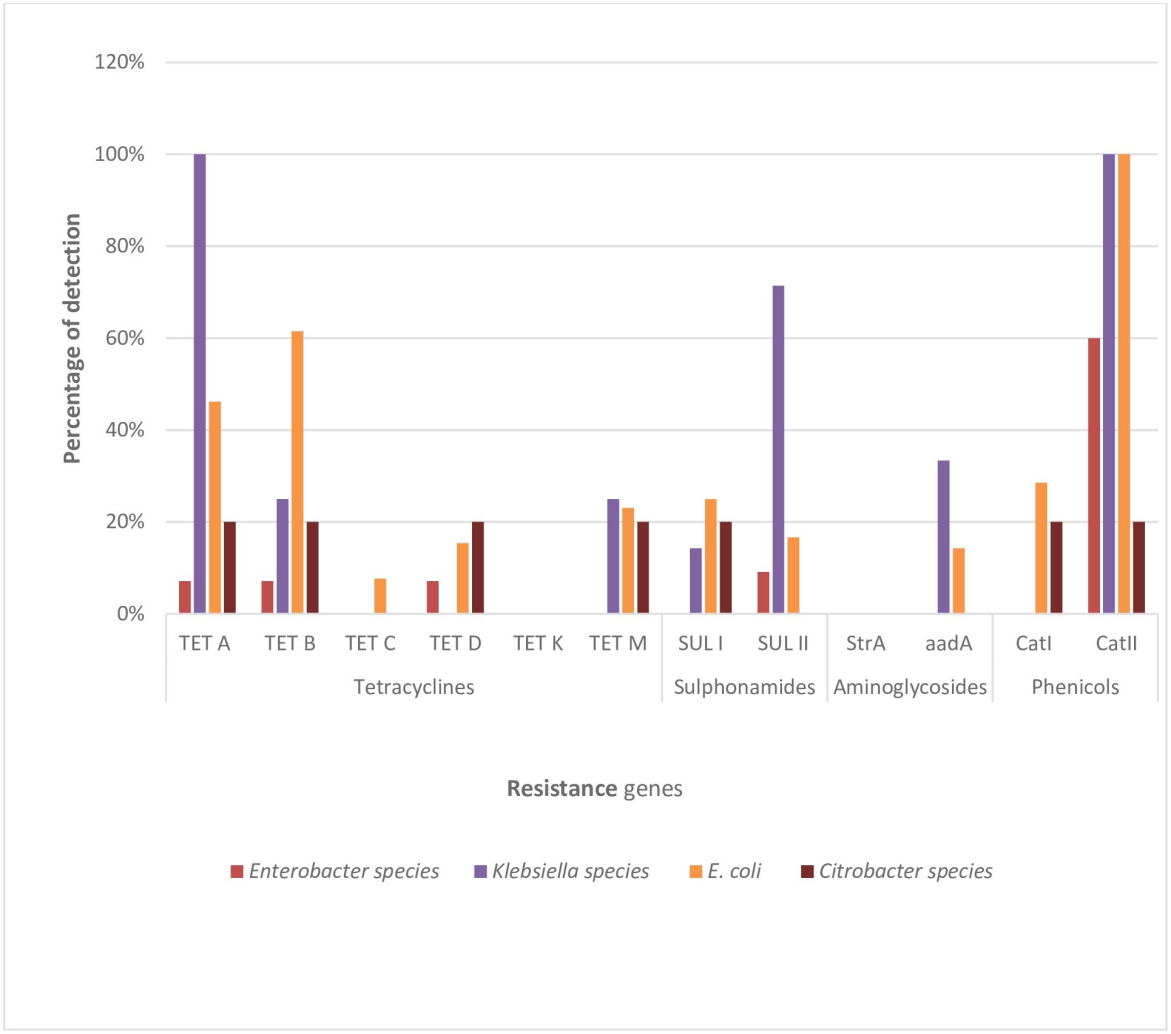
The prevalence of key non-β-lactamase resistance genes detected across the bacterial isolates. These include the genes coding for resistance against antibiotic classes tetracycline, sulphonamide, aminoglycoside, and phenicol. For tetracyclines, sulphonamides, aminoglycosides, and phenicols: *Enterobacter* spp. (n = 14, 11, 11 and 10 respectively), *Klebsiella* spp. (n = 4, 7, 3 and 3 respectively), *E*. *coli* (n = 13, 12, 7 and 7 respectively); *Citrobacter* spp. (n = 5, 5, 3 and 5 respectively) where n is the number of phenotypically resistant isolates screened for representative antibiotic resistance genes.

A variety of genes that have been implicated in antimicrobial resistance were investigated in this study. These include the three major classes of antibiotics; the β-lactams (amoxicillin/clavulanic acid and ampicillin), the carbapenems (imipenem and meropenem), and the cephems (cefotaxime and cefuroxime). Also investigated in this study are some other equally essential resistance genes which do not belong to the β-lactams. The genes include some resistance determinants for antibiotic classes sulphonamide, tetracycline, phenicol, and aminoglycoside. Almost all the 19 β-lactamase resistance genes assayed for were recovered in the members of the Enterobacteriaceae studied except one ESBL (*bla*_VEB_) and two pAmpC (*bla*_DHA_ and *bla*_MOX_) genes, which were not detected. The frequencies of detection across the bacterial isolates are as presented in Fig 6. Also, among the 12 non-β-lactamase resistance genes assayed for, only the *tetK* and *StrA gene* were not detected in this study. These represented the tetracyclines and aminoglycosides, respectively. The frequencies of detection of these genes are also depicted in Fig 7.

According to the results in Fig 6, the most prevalent resistant determinant among the β-lactams is the *bla*_TEM_ gene, with 63%, 38%, and 20% harboured in *Klebsiella* spp., *E*. *coli*, and *Citrobacter* spp., respectively. The gene *bla*_SHV_ was detected in *Klebsiella* spp., *E*. *coli*, and *Enterobacter* spp. with 50%, 23%, and 7%, respectively. At least one of the CTX-M genes (including groups 1, 2, 9) was detected across all the isolates, with *bla*_CTX-M-2_ being the most frequently detected allelic variant. *Bla*_CTX-M-2_ was detected in *E*. *coli* (38%), *Citrobacter* spp. (20%), *Klebsiella* spp. (13%), and *Enterobacter* spp. (7%). The OXA-1-like gene was only detected in *E*. *coli* (31%), while only one *Citrobacter koseri* harboured *bla*_PER_. In this category, none of the isolates harboured *bla*_VEB_. *E*. *coli* harboured most carbapenemase-resistant determinants. *Bla*_IMP_ and *bla*_VIM_ (100%), and *bla*_OXA-48-like_ (50%) ARGs were found only in *E*. *coli* while 67% of *bla*_GES_ detected was harboured by only *Citrobacter* spp. The *bla*_KPC_ gene was harboured in *E*. *coli* (100%) and *Citrobacter* spp. (33%). For pAmpC, the resistance determinants for the antibiotic class cephems, the most frequently detected was *bla*_EBC_, with 45%, 20%, and 13% being detected in *E*. *coli*, *Citrobacter* spp., and *Klebsiella* spp. Following keenly in this category is *bla*_ACC_, with 27%, 25%, and 20% in *E*. *coli*, *Klebsiella* spp., and *Citrobacter* spp. *Bla*_FOX_ was detected in *E*. *coli* (36%), *Klebsiella* spp. (13%), and *Enterobacter* spp. (7%) while *bla*_CIT_ was 20%, 13%, and 9% in *Citrobacter* spp., *Klebsiella* spp., and *E*. *coli*, respectively. None of the isolates harboured *bla*_DHA_ and *bla*_MOX_.

Among the non-β-lactamase resistance genes assayed, the most frequently detected was the *catII* encoding resistance to the class phenicol. All the *E*. *coli* and *Klebsiella* spp. recovered harboured this *catII* gene with 60% and 20% detected in *Enterobacter* spp. and *Citrobacter* spp., respectively. Meanwhile, the other gene assayed for in the phenicol resistant isolates, *catI* genes, were harboured only in *E*. *coli* and *Citrobacter* spp., isolated with 29% and 30% frequencies, respectively. In the class aminoglycosides, the *aadA* gene was harboured only in *Klebsiella* spp. (33%) and *E*. *coli* (14%) isolated while the *StrA* gene was not detected in any isolate. Among the genes assayed for tetracycline resistance, the highly detected was *tetA* with 100%, 46%, 20%, and 7% detected in *Klebsiella* spp., *E*. *coli*, *Citrobacter* spp., and *Enterobacter* spp., respectively. Other genes frequently detected were *tetB*, *tetM*, *tetD*, and *tetC* gene in that descending order. None of the isolates harboured the *tetK* gene. For the sulphonamides, the gene *sulII* was more detected with 8 of the isolates harbouring this gene than its *sulI* counterpart, which had only 5 of the isolates harbouring the gene.

Various detected genotypic patterns of the MDR bacteria isolates were also described in Table 3. The ESBLs genes detected included *bla*_CTX-M_ (including groups 1, 2, and 9), *bla*_TEM_, *bla*_VIM_, *bla*_OXA-1-like_, *bla*_OXA-48-Like_, *bla*_SHV_, *bla*_PER_, *bla*_GES_, *bla*_IMP_, and *bla*_KPC_. The pAmpC genes which were detected include *bla*_ACC_, *bla*_EBC_, *bla*_FOX_, and *bla*_CIT_. The non-β-lactam resistance genes detected include *tetA*, *tetB*, *tetC*, *tetD*. *tetM*, *sulI*, *sulII*, *aadA*, *catI*, and *catII*. Out of the 40 MDR bacteria isolates, 28 harboured at least one of the resistance genes assayed. Notably, all the genotypic patterns occurred uniquely.

**Table 3:** Extended-spectrum and plasmid-mediated AmpC β-lactamases and non-β-lactamase genotypes detected in multidrug-resistant strains.

Isolate code^c^	ESBL genes	pAmpC genes	Non-β-lactamase genes
2	*bla*_SHV_		
3			*tetA-catII*
5			*tetD-catll*
6			*tetB*
7	*bla*_FOX_		*catll*
8			*sulII*
9			*catII*
11	*bla*_CTX-M2_		*catII*
13			*catII*
15	*bla*_OXA-48-Like_-*bla*_SHV_-*bla*_CTX-M2_	*bla*_ACC_-*bla*_EBC_-*bla*_FOX_	*tetA-sulI-catII*
16	*bla*_TEM_		*sulII*
18	*bla*_TEM_		*tetA-tetM- sulII-aadA- catII*
19	*bla*_TEM_-*bla*_SHV_		*tetA- tetB- sulII*
20	*bla*_TEM_-*bla*_SHV_-*bla*_CTX-M1_		*tetA- sulII*
21	*bla*_SHV_-*bla*_CTX-M1_	*bla*_CIT_	*sulII*
22		*bla*_ACC_-*bla*_EBC_	
23	*bla*_TEM_-*bla*_GES_-*bla*_PER_- *bla*_KPC_- *bla*_CTX-M2_	*bla*_ACC_-*bla*_CIT_	*tetA- tetB- tetD- sulI-catII*
24	*bla*_GES_		
29	*bla*_TEM_-*bla*_OXA-1-like_-*bla*_IMP_-bla_VIM_-*bla*_KPC_-*bla*_CTX-M2_	*bla*_CIT_	*tetA- tetB- tetC*
30	*bla*_TEM_-*bla*_OXA-1-like_-*bla*_SHV_-*bla*_IMP_-*bla*_VIM_-*bla*_KPC_-*bla*_CTX-M2_	*bla*_CIT_-*bla*_ACC_-*bla*_EBC_-*bla*_FOX_	*tetA- tetB-tetM- catII*
31	*bla*_CTX-M2_	*bla*_EBC_-*bla*_FOX_	*tetA- tetB- catII*
32	*bla*_TEM_-*bla*_OXA-1-like_-*bla*_SHV_-*bla*_CTX-M2_	*bla*_ACC_-*bla*_EBC_	*tetA- tetB- tetM- catII*
33	*bla*_TEM_-*bla*_SHV_-*bla*_CTX-M2_-*bla*_CTX-M9_		*tetA- tetB- tetD- sulII- aadA-catII*
34	*bla*_VIM_		*tetB- sulII*
36	*bla*_TEM_-*bla*_OXA-1-like_-*bla*_CTX-M2_	*bla*_EBC_	*tetB- tetD- catII*
37			*tetA*
39		*bla*_ACC_-*bla*_EBC_-*bla*_FOX_	*tetB- catII*
40	*bla*_TEM_		*tetM*

^c^ Isolate code represents bacterial species in Fig 4.

## Discussion

The distribution of presumptive Enterobacteriaceae in the rivers sampled in this study shows that coliform bacteria are widely distributed in aquatic environments. The count range observed falls within the limit of between 100 and 100,000 CFU/ 100ml for polluted surface water [52]. A study by [53] on the bacteriological qualities of the Tyhume river suggested that the quality of the river water is poor. This corroborates the finding in this study that coliform bacteria impair the water quality. The benchmark value for faecal coliforms being 0 CFU/100ml is set in South Africa for domestic water [54], and this was employed for the interpretation of the data obtained. This is necessary because, across all sampling points, the water is being used for various domestic purposes. The detection and subsequent confirmation of the Enterobacteriaceae family members from the rivers may be attributable to the probable point and non-point pollution across the various sampling points. Observations revealed that the rivers at the sampling sites also serve as sources of drinking water for livestock and irrigation, among other purposes such as recreational (swimming) and religious (baptism) activities. It is also important to note that the rivers received wastewater runoffs from surrounding communities upstream of the sampled points. These activities highlighted could contribute substances that are capable of inducing resistance in microorganisms either directly or indirectly and could be responsible for the abundance of the various species belonging to the Enterobacteriaceae family in these water sources. Surface water sources are considered to be reservoirs of microorganisms, metals, and antibiotic resistance genes. Thereby plays a vital role in not only the spread but also in their active transport since surface water sources are recipients of wastewaters harbouring contaminants from diverse sources [5, 55–57].

The peaks of the presumptive Enterobacteriaceae were observed at T4 and TS2 for Tyhume and Tsomo rivers, respectively. It is highly pertinent to state that these sites with the highest counts are the only ones with semi-urban settlements on the different river courses. There is a probable higher pressure of anthropogenic activities around these sites, necessitating a higher than usual bacterial counts. The highest distribution of the presumptive Enterobacteriaceae observed at point T4 in Alice along the Tyhume river course could not be unconnected with the discharge from a municipal WWTP upstream and higher pressure of anthropogenic activities observed around the area during sampling. This higher than usual bacterial count obtained is relatively close to what was described by [53]. Most research concepts have employed methods that allow for the enumeration of total bacteria count irrespective of the prevailing conditions during sampling. Some studies focused on selected Enterobacteriaceae group members’ enumeration rather than assessing species’ collection in different aquatic environments. The different approaches have, however, indicated that the members of the Enterobacteriaceae group had become a common feature in much surface water globally. The presence of faecal pathogens could have a grave public health concern, especially in developing countries where dependence on surface water sources is still high due to inaccessibility to potable water for domestic and other uses. Bringing into perspective the water quality guideline, the rivers’ quality across all the sites in this study were below the standard limits, thereby making them unsuitable for domestic utilisations.

The MALDI-TOF confirmation of the identities of the presumptive Enterobacteriaceae isolated revealed that *E*. *coli* was mostly detected in this study with a 33% detection rate. A similar result was reported by [10] and [11]. However, this high detection rate of *E*. *coli* when considered with the other members of Enterobacteriaceae is unsurprising as *E*. *coli* is a common commensal organism in the gastrointestinal tracts of animals as well as humans. One of the major causes of waterborne disease outbreaks is the consumption of contaminated water, which significantly harbours gastrointestinal microbial pathogens. Rivers are generally considered as reservoirs of ARB more, mainly because they are recipients of surface waters containing materials from different origins. These sources include effluents from WWTPs, urban, and industrial effluents, which harbour an immense antibiotic resistome comprising of both pathogenic and non-pathogenic bacteria [38, 58]. The presence of ARB in rivers is a significant public health concern. This is because they can be transferred to humans when the contaminated water is consumed either directly as drinking water, or indirectly during other uses such as during recreational activities, religious activities, abstracted for irrigation, just to mention a few. The use of these rivers, in turn, can contribute to the rapid and aggravated spread and persistence of ARB within the general populace and environment. Effluents of low quality from WWTPs, hospitals, and abattoirs can easily contaminate the water-bodies that receive this discharge with potential MDR pathogenic Enterobacteriaceae species. Although this current study did not extend its investigation into the isolates’ pathogenic potential, however, all the selected Enterobacteriaceae isolates revealed a widespread presence of MDR bacteria in Tyhume and Tsomo Rivers.

Owing to the phenotypic antibiotic resistance pattern obtained, all the selected Enterobacteriaceae isolates recovered from these rivers exhibited multiple antimicrobial resistance patterns. The lowest resistance pattern being against four antibiotics, and the highest resistance pattern being against seventeen of the eighteen tested antibiotics. The majority of these resistance patterns occurred singly with three exceptions. These include a MAR phenotype pattern among *Enterobacter* spp. with resistance to fifteen antibiotics that occurred four times, a pattern among *Klebsiella* spp. and *Citrobacter* spp., each occurred twice. The breakdown of the patterns of the targeted Enterobacteriaceae isolates (40) in these rivers are as follow; four-antibiotics (2), five-antibiotics (3), seven-antibiotics (3), eight-antibiotics (1), nine-antibiotics (3), ten-antibiotics (4), eleven-antibiotics (3), twelve-antibiotics (5), thirteen-antibiotics (4), fourteen-antibiotics (4), fifteen-antibiotics (6), sixteen-antibiotics (1) and seventeen-antibiotics (1) as seen in Tables 1 and 2. This antimicrobial resistance pattern indicates that all the isolates displayed resistance against multiple antibiotics. The mean MAR index was 0.61, and the value ranged from 0.22 to 0.94, indicating heavy contamination of the rivers probably with wastewaters. The occurrence of MARPs in bacteria isolates of enteric origin found in the aquatic environment has been previously documented [9, 59–61]. In KwaZulu-Natal Province, RSA, 71.15–97.1% of *E*. *coli* isolated from two rivers in Durban exhibited MDR against the antibiotics tested [59]. Titilawo and colleagues [9] also reported multiple antimicrobial resistances ranging from three to nine antimicrobials from *E*. *coli* isolated from some rivers in Nigeria. Toroglu and colleagues [62] reported MDR in the members of Enterobacteriaceae recovered from some rivers in Turkey [62], with similar results being reported by [63] and [11] in the rivers of Bangladesh and Ethiopia, respectively. Various human activities contribute to the diverse MARPs observed in rivers. One of the most important being the discharge of wastewaters as rivers are the primary recipients of effluents from sewage [11, 64]. It is rather unfortunate that the majority of the antimicrobial agents, when used, are not fully metabolised in the body and are therefore released directly into the hospital sewage system or to the municipal wastewater [5, 23]. Final effluents of the municipal wastewater are, in turn, discharged directly into surface water. Many studies have emphasised that polluted environmental sources are mostly responsible for promoting ARGs [8, 12, 26]. The MARI of all the isolates in this study was higher than 0.2, which is an indication that these isolates were culled from high-risk environments where there is a high selective pressure of antibiotic resistance. The results obtained from this study, in general, is very close to those obtained by [65] and [66] from clinical settings wherein their isolates also exhibited high level of resistances against β-lactams, with the exception to carbapenems which are usually the last resort antibiotics for therapy against infections caused by MDR ESBL-producing Enterobacteriaceae. Likewise, a similar trend was reported by [67] for Enterobacteriaceae recovered from rivers. The aquatic environment thus remains a vital pathway/ reservoir for the spread of antimicrobial resistance in the environment.

Members of Enterobacteriaceae are natural commensals of humans and animals. Those often implicated in nosocomial and community-acquired opportunistic pathogens play a significant function in the movement of ARGs in microorganisms of environmental origin to other species and finally reaching humans, which can then be pathogenic [26, 68]. This course has been tackled by the epidemiology of disseminating ESBL/pAmpC in the aquatic milieu [26, 69–71]. Here, the genetic characterisation of essential β-lactamase genes (ESBLs and pAmpC) among β-lactam-resistant Enterobacteriaceae, which include the carbapenem-resistant Enterobacteriaceae as well as the cephem-resistant Enterobacteriaceae, revealed a high occurrence rate of various resistance determinants. The results obtained showed that *E*. *coli* and *Klebsiella* isolates harboured more of these resistance genes assayed. The higher rate of resistance observed in these two genera of Enterobacteriaceae studied could be due to their propensity to undergo stable acquisition and integration of ARGs in the aquatic environment than other species recovered. We observed twenty-eight different resistance genotype patterns in the MDR isolates, indicating that the isolates have independent clusters of genes responsible for their resistance phenotypes. The ease of the acquisition of ARGs in *E*. *coli* have been reported [40, 72]. In the context of carriage of the ESBL genes, 75% (6/8) of *Klebsiella* spp. and 62% (8/13) of *E*. *coli*, 40% (2/5) of *Citrobacter* spp., and 21% (3/14) of *Enterobacter* spp. isolates recovered harboured at least one of the genes. Based on this study, pAmpC genes were harboured in 46% (6/13) of *E*. *coli*, 25% (2/8) of *Klebsiella* spp. and 20% *Citrobacter* spp. None of the pAmpC genes was detected in any of the 14 *Enterobacter* spp. isolated.

In this study, we detected various ESBL genes, and the most predominant among the β-lactam resistance determinant was *bla*_TEM_. The dominance of bla_TEM_ could be due to their earlier emergence than other β-lactamases. *Bla*_TEM_ was the first identified enzyme that could easily hydrolyse penicillins and was commonly found in Enterobacteriaceae members [73]. The presence of *bla*_TEM_ is an indication that the selective pull favouring the ESBL genes are present in aquatic environments in South Africa. In support of our findings, the dominance of *bla*_TEM_ in *E*. *coli* has been reported in aquatic environments [9, 74]. Although the most widespread enzymes among the ESBLs are the CTX-M family [72, 75]. In this study, the next frequently detected β-lactam resistance determinant was the CTX-M subtypes (including groups 1, 2, and 9). The CTX-M-2 was more predominant among the CTX-M allelic variants and was harboured mostly in *E*. *coli* recovered. The CTX-M-2 variant is one of the most prevalent in South Africa [76, 77]. These CTX-M subtypes (groups 1, 2 and 9) reported in this study have also been reported in similar studies to be the most prevalent ESBLs in *E*. *coli* isolated from aquatic and clinical settings [78–81], human faecal isolates [82], and sewage sludge [83]. The *bla*_CTX-M_ genes that keenly followed highlight the widespread of these genes in hospital and community settings in the last decade [84]. CTX-M genes’ dominance in many different surface water has been reported [39, 85, 86].

This study reports detecting the highest resistance rate among *E*. *coli* isolates to the various antibiotics classes, mainly to major β-lactams and tetracyclines. All the isolates were phenotypically resistant to these antibiotics. Consequently, a high rate of detection of the assayed ARGs detected among these isolates was not unexpected. Out of all the members of the Enterobacteriaceae isolates investigated, the β-lactamases, including the ESBLs and pAMPCs, and *tet* genes were harboured by most of the *E*. *coli* isolates with a very high detection rate of these resistance genes. As obtained in this study, high rates of resistance among *E*. *coli* strains to β-lactams and tetracyclines from river water has been reported in similar studies [9, 11, 59]. The production of β-lactamase is the primary mechanism of resistance against β-lactam antibiotics. These β-lactamases are carried on various mobile genetic elements such as plasmids, contributing to the horizontal transmission of resistance genes among other bacterial species present in the water bodies [73, 87]. Only the *E*. *coli* isolates harboured the *bla*_IMP_, *bla*_VIM_, and *bla*_OXA-48-Like_. The detection of various resistance genes could be attributed to reports that *E*. *coli* has excellent ease of acquiring resistance genes from other microorganisms [88]. This study shows that *E*. *coli* is more affected by the acquisition and stable integration of resistance genes in an aquatic environment than other Enterobacteriaceae members.

All the *Klebsiella* spp. isolated were phenotypically resistant to antibiotics belonging to β-lactams and cephems used in this study. A large proportion (88%) was also resistant to the sulfonamide, trimethoprim-sulfamethoxazole. A similar rate of resistance among the β-lactams and sulphonamide in this study has been previously reported [11]. Notably, the isolates were multidrug-resistant to several clinically important antibiotics except for reserved ones such as the carbapenems. The presence of these MDR strains, especially *K*. *pneumoniae*, from freshwater sources frequently being utilised for domestic purposes is particularly a worrying finding. As expected, the strains’ phenotypic resistance characteristics somewhat correlated with known genetic mediators of resistance. The only *K*. *oxytoca* isolate harboured only *bla*_ACC_ and *bla*_EBC_ resistance genes. This isolate was fully phenotypically resistant to β-lactams, polymyxins, and nitrofurantoin antibiotic classes and displayed intermediate resistance to one of the cephems, cefuroxime. The remaining isolates, *K*. *pneumoniae*, showed varying resistance profiles and mostly harboured *bla*_TEM_ and *bla*_SHV_ ARGs. The presence of these key ESBL genes in clinical and environmental contexts have been previously described among *Klebsiella* spp. in South African and international isolates [89–92]. Since the isolates did not display full resistance to carbapenems and consequently did not harbour any of the carbapenemases genes, it was assumed that these could be kept as reserve drugs to treat infections caused by *Klebsiella* spp.

The Enterobacter genus is also claimed to serve as a reservoir of ARGs. There are several reports regarding their ability to acquire various mobile genetic elements that can confer certain fitness advantages, enabling them to colonise several environments and hosts. In the last decade, *Enterobacter* spp. has emerged as the third most common Enterobacteriaceae resistant to third-generation cephalosporins with *E*. *coli* and *Klebsiella* spp. [93]. From the results of this study, *Enterobacter* spp. is the third most prevalent in terms of frequency of occurrence and frequency of detection of the resistance genes. There are reports of two of the well-known species, *E*. *aerogenes* and *E*. *cloacae*, responsible for the outbreaks of opportunistic nosocomial infections [93]. The *Enterobacter* spp. isolated in this study belong to four species. The most prevalent species isolated were *E*. *aerogenes* and *E*. *cloacae*, similar to what has been reported even among clinical isolates [93]. The prevalence of these MDR isolates, albeit in environmental waters, is quite concerning. It shows that these water sources are being polluted with potentially pathogenic outbreak-causing microorganisms of clinical origin. All the isolates were phenotypically resistant to β-lactams, cephems, and tetracyclines. The phenotypes observed further alludes to the reports that *Enterobacter* spp. are intrinsically resistant to ampicillin, amoxicillin-clavulanic acid, cephalothin, and cefoxitin [93, 94]. Surprisingly, they did not harbour most of the ARGs assayed in this study. Among the ESBL determinants, only three genes were detected and were distributed among three different species. One *E*. *cloacae* isolate harboured *bla*_*SHV*_, one *E*. *asburiae* harboured *bla*_FOX_, and one *E*. *aerogenes* harboured *bla*_CTX-M-2_. The only *E*. *amnigenus* isolated did not harbour any of the resistance gene determinants. This seemingly low rate of detection of ARGs is similar to a previous report [95].

Over the past decade, *Citrobacter* spp. have become resistant to the most widely used antimicrobials, such as ampicillin, ceftazidime, cefotaxime, aminoglycoside, and tetracycline [96]. This report supports this study’s findings where the *C*. *freundii* and *C*. *koseri* isolated were all resistant to a minimum of ten out of the eighteen antibiotics assayed. All the isolates were phenotypically resistant to ampicillin, amoxicillin/clavulanic acid, cefotaxime, cefuroxime, nitrofurantoin, polymyxin B, colistin sulfate, doxycycline, and tetracycline. There have been few reports on *Citrobacter* spp. in freshwater sources [96]. The isolates in this present study harboured various ARGs, as shown in Figs 6 and 7. The detection of various β-lactamases is similar to previous clinical and environmental studies [77, 95, 96]. There is a rising trend of resistance in *Citrobacter* spp. against different antibiotics commonly prescribed by physicians to treat infections caused by *Citrobacter* spp.

It is important to highlight that the simultaneous occurrence of ESBLs and pAmpCs was seen in 20% of the overall isolates recovered in this study. More importantly, the ESBL/pAmpC genes’ co-carriage was most prevalent in *E*. *coli* with a 75% occurrence. The co-occurrence of ESBL/pAmpC in *E*. *coli* correlated conspicuously with the result of another study that reported the distribution of β- lactamases genes in irrigation water in South Africa [76]. The most frequently detected genes were observed in one *E*. *coli* isolate, which harboured thirteen genes, including five ESBLs, four pAmpCs, and four non-β-lactamase genes. However, notably among the isolates was a *C*. *koseri*, which harboured twelve out of the genes assayed. The resistance genes detected include five of the ESBL genes (including the only *bla*_PER_ detected in this study), two of the pAmpC resistance genes, and five non-β-lactamase genes. This study provides the first report of C. koseri, exhibiting co-occurrence of ESBL/pAmpC in environmental isolates to the best of our knowledge. The detection of a *bla*_PER_ producing *Citrobacter* spp. in this study is impressive. This emerging ESBL gene has not been previously reported in environmental isolates in South Africa. Although the *bla*_PER_ ESBL gene has been reported in *Aeromonas* from European surface waters and Austrian activated sludge, this enzyme is rarely reported in clinical isolates worldwide [97–99]. It is interesting to note that Carbapenemase-Producing Enterobacteriaceae (CPE) isolates *bla*_KPC_, *bla*_GES_, *bla*_OXA-48-like_, and other variants *bla*_VIM_, *bla*_IMP_ that are often detected in clinical settings were also recovered in this present study. The *bla*_KPC_, *bla*_GES_, and *bla*_*O*XA-48-like_ have been found in clinical settings in South Africa [100]. Although it is unclear where these CPE isolates have emerged from, whether from humans or hospital settings or municipal discharges, what remains apparent is that these isolates may spread from the aquatic environment to clinical settings in the most foreseeable future. The detection of carbapenemase and ESBL genes in rivers is worrisome and indicates that the dissemination of resistant strains to the environment is currently going on in South Africa.

A high prevalence of non-β-lactamases was also detected in this study. This includes *tetA* (33.3%), *tetB* (30.5%), *tetM* (13.9%), *tetD* (11.1%), *tetC* (2.8%), *catII* (68%), *catI* (12%), *sulII* (22.9%), *sulI* (14.3%), and *aadA* (8.3%). Most of these isolates recovered from these rivers harboured multiple antibiotic resistance genes, which include the ESBLs, pAmpC, as well as the non-β-lactamases. Combining these genes in MDR Enterobacteriaceae can potentially result in disastrous effects when transferred into humans directly while utilising these water sources for various domestic purposes or indirectly when the water is abstracted for irrigation of fresh produce, which usually require minimal or no processing before consumption.

## Conclusion

The findings from this study exposed the high occurrence of MDR Enterobacteriaceae in Tsomo and Tyhume rivers, thus requiring special attention to not compromise the water-food-public health interphase. The high frequency of detecting the various ARGs investigated indicates that the MARP observed is mostly due to these genes that confer resistance against various antibiotics covered in this study. ARGs widely found in Enterobacteriaceae have been established to be harboured on various mobile genetic elements that play a critical function in the rapid spread of ARG determinants. Our findings support the need for comprehensive control of ARG dissemination into the environment. Controlling the dissemination of ARGs is necessary due to the serious public health concern attached to the proliferation of microbial pathogens into the environment and the menace of antibiotic resistance. Some control can be achieved by proper management of antibiotic use in human and animal husbandry, effective management of waste (hospital, municipal, agricultural, and industrial) containing antimicrobial resistance bacteria and ARGs. Lastly, the management of already contaminated environments. All of the strategies mentioned above are important in forestalling the scourge of antimicrobial resistance spread in the environment.

## Supporting information

S1 File (DOCX)

S1 Graphical abstract (TIF)
