# Supplementary material for: Occurrence, identification, and antibiogram signatures of selected Enterobacteriaceae from Tsomo and Tyhume rivers in the Eastern Cape Province, Republic of South Africa
Source: PLoS One. 2020 Dec 7;15(12):e0238084. doi: 10.1371/journal.pone.0238084 (PMC7721149; doi:10.1371/journal.pone.0238084)
Supplement: S1 File — (DOCX) [file pone.0238084.s001.docx]

**Supplementary Table 1:** Description of sampling points.

| **District Municipality** | **Sampling site** | **Site Code** | **Description of site** | **Geographical coordinates** |
| --- | --- | --- | --- | --- |
| Amathole | Hala | T 1 | This is a rural community located immediately downstream the source of the Tyhume river which is located in Hogsback. | 32°36ʹ39ʺ S and 26°54ʹ34ʺ E |
|  | Khayalethu | T 2 | Khayalethu is a major rural community located upstream the Binfield Park Dam. This river is very important to the inhabitants of this community as it is used for irrigation, recreation, drinking, and other domestic activities. | 32°38ʹ22ʺ S and 26°51ʹ27ʺ E |
|  | Sinakanaka | T3 | It is a rural community found on the banks of the Tyhume river which is further downstream of Khayalethu with several densely populated settlements. The water from this river is utilized by the inhabitants of these settlements for various activities such as drinking, fishing, irrigation, recreation, as well as other domestic purposes. | 32°45ʹ37ʺ S and 26°51ʹ27ʺ E |
|  | Alice | T4 | This is a semi-urban settlement with many subunits. In the western axis is Happy Rest community. Gaga, Gqumashe, and Ntselemanzi are located to the north-west while Golf Course is located to the north. This site also harbours the student population of the University of Fort Hare to the east. The river is used extensively for irrigation, fishing, domestic purposes, and as a source of drinking water for livestock. Municipal wastewater effluent discharge was observed upstream. | 32°47ʹ17ʺ S and 26°50ʹ31ʺ E |
| Chris Hani | Bongweni | TS1 | Bongweni Village is a rural settlement where the Tsomo river is used extensively for domestic and agricultural purposes. The river merges with the Ngcongcolora river and empties into the Great Kei River. | 32°4ʹ50ʺ S and 27° 47ʹ 9ʺ E |
|  | Tsomo | TS2 | This is a semi-urban settlement with many subunits. The apparent use of the water at the point of sampling includes washing, religious activities and source of water for farm animals. The river is used extensively for irrigation, fishing, domestic purposes, and as a source of drinking water downstream of the sampling point. | 32°2ʹ38ʺ S and 27°49ʹ19ʺ E |

Note: For confidentiality sake, sampling sites are coded. All the information recorded in this table were derived onsite. Coordinates for each sampling site were retrieved using the “etrex-LEGENDH” GPS equipment.

**Supplementary Table 2:** Primers used for targeting resistances in β-lactams, carbapenems and cephems (Dallenne et al., 2010).

| **PCR name** | **β-Lactamase(s) targeted** | **Primer name** | **Sequence (5ʹ –3ʹ)** | **Amplicon**  **size (bp)** |
| --- | --- | --- | --- | --- |
| Multiplex I TEM, SHV and OXA-1-like | TEM variants including TEM-1 and TEM-2 | MultiTSO-T_for  MultiTSO-T_rev | CATTTCCGTGTCGCCCTTATTC CGTTCATCCATAGTTGCCTGAC | 800 |
|  | SHV variants including SHV-1 | MultiTSO-S_for  MultiTSO-S_rev | AGCCGCTTGAGCAAATTAAAC ATCCCGCAGATAAATCACCAC | 713 |
|  | OXA-1, OXA-4 and OXA-30 | MultiTSO-O_for  MultiTSO-O_rev | GGCACCAGATTCAACTTTCAAG GACCCCAAGTTTCCTGTAAGTG | 564 |
| Multiplex II CTX-M group 1, group 2 and group 9 | variants of CTX-M group 1 including  CTX-M-1, CTX-M-3 and CTX-M-15 | MultiCTXMGp1_for  MultiCTXMGp1-2_rev | TTAGGAARTGTGCCGCTGYA^b^ CGATATCGTTGGTGGTRCCAT^b^ | 688 |
|  | variants of CTX-M group 2 including CTX-M-2 | MultiCTXMGp2_for  MultiCTXMGp1-2_rev | CGTTAACGGCACGATGAC CGATATCGTTGGTGGTRCCAT^b^ | 404 |
|  | variants of CTX-M group 9 including  CTX-M-9 and CTX-M-14 | MultiCTXMGp9_for  MultiCTXMGp9_rev | TCAAGCCTGCCGATCTGGT TGATTCTCGCCGCTGAAG | 561 |
| Multiplex III ACC, FOX,  MOX, DHA, CIT and EBC | ACC-1 and ACC-2 | MultiCaseACC_for  MultiCaseACC_rev | CACCTCCAGCGACTTGTTAC GTTAGCCAGCATCACGATCC | 346 |
|  | FOX-1 to FOX-5 | MultiCaseFOX_for  MultiCaseFOX_rev | CTACAGTGCGGGTGGTTT CTATTTGCGGCCAGGTGA | 162 |
|  | MOX-1, MOX-2, CMY-1, CMY-8 to CMY-11 and CMY-19 | MultiCaseMOX_for  MultiCaseMOX_rev | GCAACAACGACAATCCATCCT GGGATAGGCGTAACTCTCCCAA | 895 |
|  | DHA-1 and DHA-2 | MultiCaseDHA_for  MultiCaseDHA_rev | TGATGGCACAGCAGGATATTC GCTTTGACTCTTTCGGTATTCG | 997 |
|  | LAT-1 to LAT-3, BIL-1, CMY-2 to MY-7,  CMY-12 to CMY-18 and CMY-21 to CMY-23 | MultiCaseCIT_for  MultiCaseCIT_rev | CGAAGAGGCAATGACCAGAC ACGGACAGGGTTAGGATAGY^b^ | 538 |
|  | ACT-1 and MIR-1 | MultiCaseEBC_for  MultiCaseEBC_rev | CGGTAAAGCCGATGTTGCG AGCCTAACCCCTGATACA | 683 |
| Multiplex IV VEB, PER and GES | GES-1 to GES-9 and GES-11 | MultiGES_for  MultiGES_rev | AGTCGGCTAGACCGGAAAG TTTGTCCGTGCTCAGGAT | 399 |
|  | PER-1 and PER-3 | MultiPER_for  MultiPER_rev | GCTCCGATAATGAAAGCGT TTCGGCTTGACTCGGCTGA | 520 |
|  | VEB-1 to VEB-6 | MultiVEB_for  MultiVEB_rev | CATTTCCCGATGCAAAGCGT CGAAGTTTCTTTGGACTCTG | 648 |
| Multiplex V GES and OXA-48-like | GES-1 to GES-9 and GES-11 | MultiGES_for  MultiGES_rev | AGTCGGCTAGACCGGAAAG TTTGTCCGTGCTCAGGAT | 399 |
|  | OXA-48-like | MultiOXA-48_for  MultiOXA-48_rev | GCTTGATCGCCCTCGATT GATTTGCTCCGTGGCCGAAA | 281 |
| Multiplex VI IMP, VIM and KPC | IMP variants except IMP-9, IMP-16,  IMP-18, IMP-22 and IMP-25 | MultiIMP_for  MultiIMP_rev | TTGACACTCCATTTACDG^b^ GATYGAGAATTAAGCCACYCT^b^ | 139 |
|  | VIM variants including VIM-1 and VIM-2 | MultiVIM_for^c^  MultiVIM_rev^c^ | GATGGTGTTTGGTCGCATA CGAATGCGCAGCACCAG | 390 |
|  | KPC-1 to KPC-5 | MultiKPC_for  MultiKPC_rev | CATTCAAGGGCTTTCTTGCTGC ACGACGGCATAGTCATTTGC | 538 |

^a^Annealing position within the corresponding open reading frame (from the base A of start codon ATG).

^b^Y¼T or C; R¼A or G; S¼G or C; D¼A or G or T.

^c^This primer pair was previously described by Ellington et al. (2007).

**Supplementary Table 3:** List of primers used in targeting resistances in non-β-lactam antimicrobial classes.

| **Antimicrobial family** | **Primer name** | **Primer sequence (5ʹ-3ʹ)** | **Amplicon size (bp)** | **Reference** |
| --- | --- | --- | --- | --- |
| Sulfonamides | *sul I* | F:TTCGGCATTCTGAATCTCAC | 822 | Maynard et al. (2004) |
|  |  | R:ATGATCTAACCCTCGGTCTC |  |  |
|  | *sul II* | F:CGGCATCGTCAACATAACC | 625 | Falbo et al. (1999) |
|  |  | R:GTGTGCGGATGAAGTCAG |  |  |
| Tetracyclines | *tetA* | F:GCTACATCCTGCTTGCCTTC | 201 | Ng et al. (2001) |
|  |  | R:CATAGATCGCCGTGAAGAGG |  |  |
|  | *tetB* | F: TTGGTTAGGGGCAAGTTTTG | 359 | Ng et al. (2001) |
|  |  | R:GTAATGGGCCAATAACACCG |  |  |
|  | *tetC* | F:CTTGAGAGCCTTCAACCCAG | 418 | Ng et al. (2001) |
|  |  | R:ATGGTCGTCATCTACCTGCC |  |  |
|  | *tetD* | F:AAACCATTACGGCATTCTGC | 300 | Ng et al. (2001) |
|  |  | R:GACCGGATACACCATCCATC |  |  |
|  | *tetK* | F:GTAGCGACAATAGGTAATAGT | 460 | Strommenger et al. (2003) |
|  |  | R:GTAGTGACAATAAACCTCCTA |  |  |
|  | *tetM* | F:AGTGGAGCGATTACAGAA | 158 | Strommenger et al. (2003) |
|  |  | R:CATATGTCCTGGCGTGTCTA |  |  |
| Phenicols | *catI* | F:AGTTGCTCAATGTACCTATAACC | 320 | Maynard et al. (2004) |
|  |  | R:TTGTAATTCATTAAGCATTCTGCC |  |  |
|  | *catII* | F:ACACTTTGCCCTTTATCGTC | 543 | Maynard et al. (2004) |
|  |  | R:TGAAAGCCATCACATACTGC |  |  |
| Aminoglycosides | *strA* | F:CTTGGTGATAACGGCAATTC | 348 | Velusamy et al. (2007) |
|  |  | R:CCAATCGCAGATAGAAGGC |  |  |
|  | *aadA* | F:GTGGATGGCGGCCTGAAGCC | 525 | Velusamy et al. (2007) |
|  |  | R:AATGCCCAGTCGGCAGCG |  |  |

**Supplementary Table 4:** MALDI-TOF identification of presumptive isolates obtained from the rivers.

| **Family** | **Genus** | **Species** | **Number identified** |
| --- | --- | --- | --- |
| Bacillaceae | *Bacillus* | *pumilus* | **2** |
|  |  |  |  |
| Enterobacteriaceae | *Citrobacter* | *freundii* | 4 |
| Enterobacteriaceae | *Citrobacter* | *koseri* | 1 |
| Enterobacteriaceae | *Enterobacter* | *aerogenes* | 7 |
| Enterobacteriaceae | *Enterobacter* | *amnigenus* | 1 |
| Enterobacteriaceae | *Enterobacter* | *asburiae* | 2 |
| Enterobacteriaceae | *Enterobacter* | *cloacae* | 4 |
| Enterobacteriaceae | *Escherichia* | *coli* | 13 |
| Enterobacteriaceae | *Escherichia* | *hermannii* | 3 |
| Enterobacteriaceae | *Klebsiella* | *oxytoca* | 1 |
| Enterobacteriaceae | *Klebsiella* | *pneumoniae* | 7 |
| Enterobacteriaceae | *Plesiomonas* | *shigelloides* | 1 |
| Enterobacteriaceae | *Proteus* | *mirabilis* | 6 |
| Enterobacteriaceae | *Proteus* | *vulgaris* | 2 |
| Enterobacteriaceae | *Serratia* | *marcescens* | 4 |
|  |  |  | **56** |
|  |  |  |  |
| Morganellaceae | *Providencia* | *alcalifaciens* | **1** |
|  |  |  |  |
| Pseudomonadaceae | *Pseudomonas* | *corrugata* | 1 |
| Pseudomonadaceae | *Pseudomonas* | *fragi* | 1 |
| Pseudomonadaceae | *Pseudomonas* | *koreensis* | 1 |
| Pseudomonadaceae | *Pseudomonas* | *monteilii* | 2 |
|  |  |  | **5** |
|  |  |  |  |
| Staphylococcaceae | *Staphylococcus* | *sciuri* | **2** |
|  |  |  |  |
| Not reliable for identification | | | **13** |
|  |  |  |  |
| **GRAND TOTAL** | | | **79** |

**Figure 2:** The distribution of the confirmed members of the targeted Enterobacteriaceae (n=40) recovered from the rivers.

**REFERENCES**

**Dallenne, C., Da Costa, A., Decre´, A., Favier, C., & Arlet, G. 2010.** Development of a set of multiplex PCR assays for the detection of genes encoding important b-lactamases in Enterobacteriaceae. *J. Antimicrob. Chemother.* 65: 490–495.

**Ellington, M.J., Kistler, J., Livermore, D.M. et al. 2007**. Multiplex PCR for rapid detection of genes encoding acquired metallo-b-lactamases. *J. Antimicrob. Chemother.* 59: 321–322.

**Falbo, V., Carattoli, A., Tosini, F., Pezzella, C., Dionisi, A.M., & Luzzi, I. 1999.** Antibiotic resistance conferred by a conjugative plasmid and a class I integron in *Vibrio cholerae* O1 El Tor strains isolated in Albania and Italy. *Antimicrob. Agents Chemother*. 43, 693–696.

**Maynard, C., Bekal, S., Sanschagrin, F., Levesque, R.C., Brousseau, R., Masson, L., Lariviere, S., & Harel, J. 2004.** Heterogeneity among virulence and antimicrobial resistance gene profiles of extraintestinal *Escherichia coli* isolates of animal and human origin. *J. Clin. Microbiol*. 42 (12), 5444–5452.

**Ng, L.K., Martin, I., Alfa, M., & Mulvey, M. 2001.** Multiplex PCR for the detection of tetracycline resistant genes*. Mol. Cell. Probes* 15: 209–215.

**Strommenger, B., Kettlitz, C., Werner, G., & Witte, W. 2003.** Multiplex PCR assay for simultaneous detection of nine clinically relevant antibiotic resistance genes in *Staphylococcus aureus*. *J. Clin. Microbiol*. 41, 4089–4094.

**Velusamy, S., Barbara, E.G., Mark, J.L., Lien, T.N., Susan, I.H., Ynte, H.S., & Stephen, P.O. 2007.** Phenotypic and genotypic antimicrobial resistance patterns of *Escherichia coli* isolated from dairy cows with mastitis. *Vet. Microbiol*. 124: 319–328.
