# Supplementary figures and images for: Occurrence, identification, and antibiogram signatures of selected Enterobacteriaceae from Tsomo and Tyhume rivers in the Eastern Cape Province, Republic of South Africa
Source: PLoS One. 2020 Dec 7;15(12):e0238084. doi: 10.1371/journal.pone.0238084 (PMC7721149; doi:10.1371/journal.pone.0238084)

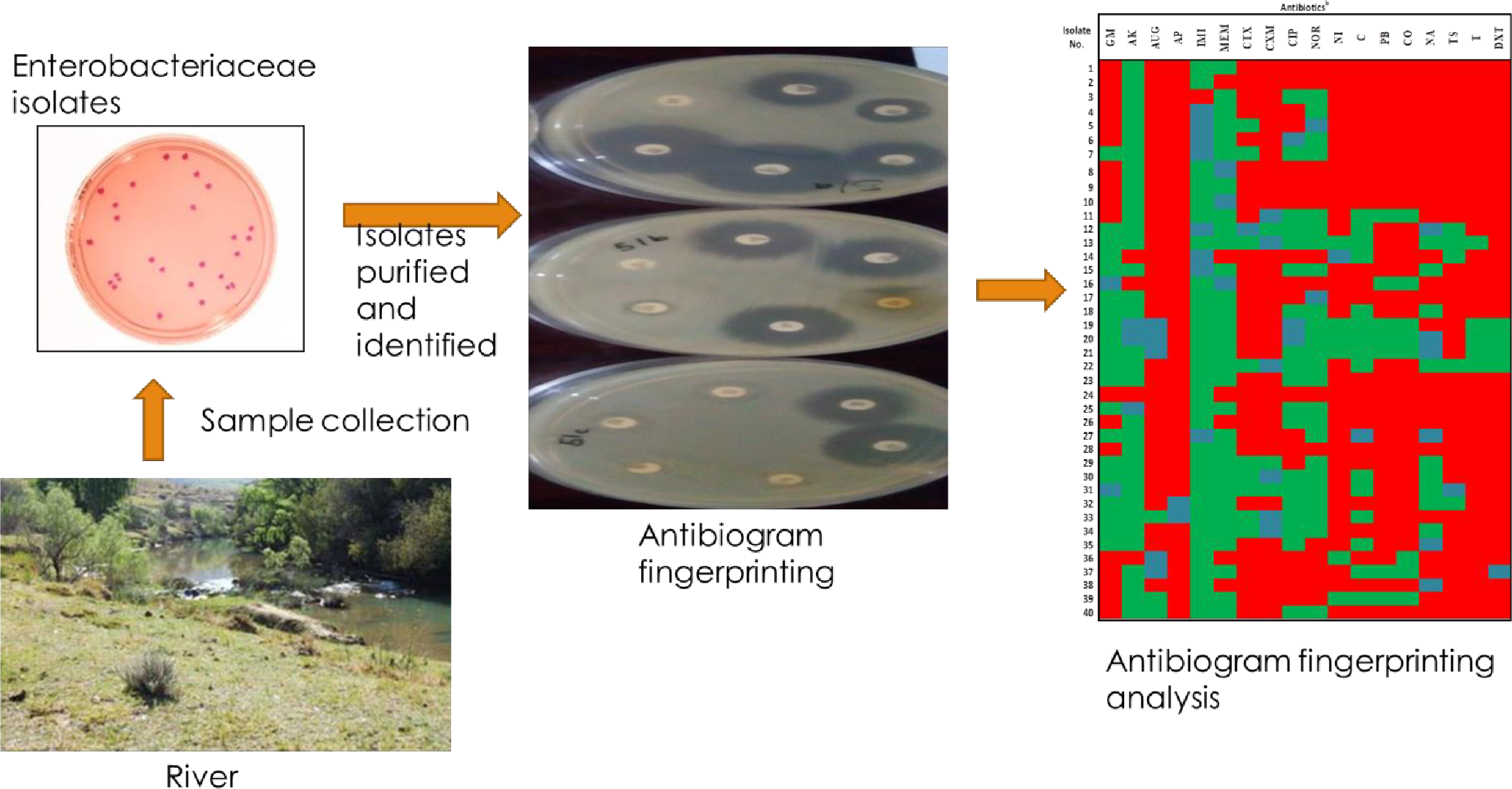

Supplement: S1 Graphical abstract — (TIF) [file pone.0238084.s002.tif]
